# EJE AWARD 2019: New diagnostic approaches for patients with polyuria polydipsia syndrome

**DOI:** 10.1530/EJE-19-0163

**Published:** 2019-05-08

**Authors:** Mirjam Christ-Crain

**Affiliations:** 1 Department of Endocrinology, Diabetology and Metabolism and Department of Clinical Research, University Hospital Basel, Basel, Switzerland; 2 University of Basel, Basel, Switzerland

## Abstract

Diabetes insipidus (DI), be it from central or nephrogenic origin, must be differentiated from secondary forms of hypotonic polyuria such as primary polydipsia. Differentiation is crucial since wrong treatment can have deleterious consequences. Since decades, the gold standard for differentiation has been the water deprivation test, which has limitations leading to an overall unsatisfying diagnostic accuracy. Furthermore, it is cumbersome for patients with a long test duration. Clinical signs and symptoms and MRI characteristics overlap between patients with DI and primary polydipsia. The direct test including vasopressin (AVP) measurement upon osmotic stimulation was meant to overcome these limitations, but failed to enter clinical practice mainly due to technical constraints of the AVP assay. Copeptin is secreted in equimolar amount to AVP but can easily be measured with a sandwich immunoassay. A high correlation between copeptin and AVP has been shown. Accordingly, copeptin mirrors the amount of AVP in the circulation and has led to a ‘revival’ of the direct test in the differential diagnosis of DI. We have shown that a baseline copeptin, without prior thirsting, unequivocally identifies patients with nephrogenic DI. In contrast, for the differentiation between central DI and primary polydipsia, a stimulated copeptin level of 4.9 pmol/L upon hypertonic saline infusion differentiates these two entities with a high diagnostic accuracy and is superior to the water deprivation test. Close sodium monitoring during the test is a prerequisite. Further new test methods are currently evaluated and might provide an even simpler way of differential diagnosis in the future.

## Invited Author’s profile


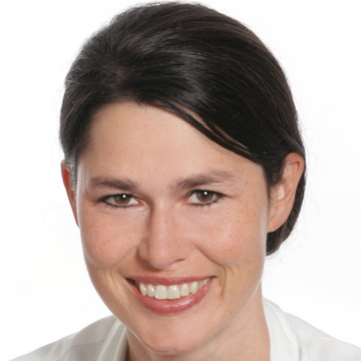


**Mirjam Christ-Crain** is full Professor of Endocrinology, Diabetes and Metabolism at the University of Basel and the University Hospital of Basel, Switzerland. From 2013 she has been the Deputy Chief at the Clinic for Endocrinology, University Hospital Basel and from 2014 she has been the Head of the Department of Clinical Research at the University of Basel. Her main research interest is on vasopressin-dependent disorders of fluid homeostasis, i.e. DI and hyponatremia. She has authored and co-authored more than 200 publications and received several awards for her research.

## Introduction

DI (DI) belongs to the polyuria polydipsia syndrome and is characterized by a high urinary output of more than 50 mL per kg body weight per 24 h, accompanied by polydipsia of more than 3 L a day (1). After exclusion of AVP-independent causes (such as hyperglycemia), the differential diagnosis of hypotonic polyuria includes central or nephrogenic DI and primary polydipsia. Differentiation is crucial since treatment varies and wrong treatment can have dangerous consequences.

Since decades, the gold standard for differential diagnosis is the classical indirect water deprivation test, which however, has its limitations and has a diagnostic accuracy of only around 70%. To overcome these limitations, the direct test was proposed with measurement of arginine vasopressin (AVP) upon osmotic stimulation. However, despite initial promising results, this test did not enter clinical routine, mainly due to technical limitations of the AVP assay. Novel approaches are therefore urgently needed. This review focuses on new diagnostic approaches in the differential diagnosis of polyuria polydipsia syndrome.

## The polyuria polydipsia syndrome

Polyuria polydipsia syndrome is a common problem in clinical practice with the main entities being central or nephrogenic DI and primary polydipsia (2).

Central DI is characterized by an insufficient vasopressin (AVP) secretion from the pituitary (3, 4), and nephrogenic DI results from an AVP resistance at the level of the kidneys (5). Both entities lead to hypotonic polyuria with consequent polydipsia. The most common form of central DI occurs mainly due to lesions of the posterior pituitary or the hypothalamic median eminence. A number of acquired disorders (e.g. trauma, neoplastic disease, granulomatous diseases) can lead to central DI. Transsphenoidal surgery can lead to transient central DI in up to 30% of cases and to permanent central DI in 2–10% (6). Genetic defects in the AVP synthesis can lead to inherited forms of central DI (7, 8). In most cases thirst mechanisms are intact, which leads to compensatory polydipsia. However, in a variant of central DI called osmoreceptor dysfunction, thirst is also impaired and hypodipsia can result in serious complications associated with hyperosmolality (2, 9).

Nephrogenic DI results due to a lack of aquaporin 2 (AQP2)-mediated water reabsorption in the collecting duct. This can either result from mutations in the genes encoding for the key proteins *AVPR2* and *AQP2* or is secondarily triggered by biochemical disorders or by certain drugs especially lithium (5).

Excessive fluid intake over an extended period of time also causes polyuria despite intact AVP secretion and renal response, but the pathomechanism is less clear. In few cases, it can result from an abnormality in thirst mechanisms (sometimes called dipsogenic DI), but more often it is due to psychiatric disorders (referred to as psychognic polydipsia). Excessive fluid intake results in a decrease in osmolality and suppressed AVP release. Consequently, water is excreted to compensate for the high fluid intake. An analysis of 23 patients with profound hyponatremia due to primary polydipsia revealed an increased prevalence for psychiatric diagnoses such as dependency disorders (43%) and depression (35%) (10). Another prospective study with 156 patients with polyuria polydipsia syndrome however showed a similar rate of 27% for psychiatric disorders between patients with primary polydipsia and patients with complete central DI (11). As the chronic polydipsia in patients with primary polydipsia leads to a downregulation of the AQP2 channels in the kidneys, the renal medullary concentration gradient is reduced, making any diagnostic evaluation of the urinary measures difficult (12).

## Differential diagnosis of polyuria polydipsia syndrome: a historic perspective

Differentiation between the three mentioned entities is important since treatment strategies vary and application of the wrong treatment can be dangerous (13). However, reliable differentiation is often difficult to achieve (14), as available tests were unsatisfactory (15) and often resulted in false diagnoses, especially in patients with primary polydipsia or partial, mild forms of DI (1, 16).

### Clinical manifestations

Patients with DI, especially those with osmoreceptor defect syndromes, can manifest with dehydration and hyperosmolality, if water loss cannot be compensated by fluid intake. Manifestations may range from non-specific symptoms such as irritability and cognitive dysfunction to more severe manifestations such as disorientation, reduced consciousness, seizure, coma, focal neurologic deficits and cerebral infarction (17). However, most patients have an intact thirst perception and water availability. In these patients the characteristic clinical symptom is polyuria and polydipsia which does not differ in manifestation between central or nephrogenic DI or primary polydipsia.

Patients with central DI are reported to have more often nocturia and a sudden onset of symptoms, resulting from the fact that urinary concentration can be maintained until the residual capacity of the hypothalamus to synthesize AVP falls below 10–15% of normal, after which urine output increases significantly. In addition, historically, it has been reported that patients with DI in contrast to patients with primary polydipsia prefer cold water as best quenching beverage and have a more sustained and less fluctuating course of symptoms (13). However, we have only recently prospectively evaluated clinical signs and symptoms: Although the majority of patients with DI indeed reported a sudden onset of symptoms, still more than one-third experienced a slow process. Moreover, also more than 60% of patients with primary polydipsia reported nightly drinking, the majority of them preferred cold beverages, and nearly 80% indicated a sustained character of symptoms. Interestingly and in contrast with the described high prevalence of psychiatric diseases in primary polydipsia, less than 30% of patients with primary polydipsia had been psychiatrically diagnosed, which was the same prevalence as found in patients with complete central DI (11). Taken together, clinical signs and symptoms are not specific and sensitive enough to differentiate between the various entities of the polyuria polydipsia syndrome.

### Radiological findings

Unenhanced brain MRI via assessment of the posterior pituitary and the pituitary stalk has been reported to provide important information in the differential diagnosis of DI. Specifically, an area of hyperintensivity, referred to as the pituitary ‘bright spot’, is normally observed in the posterior part of the sella turcica in sagittal views on T1-weighted images (18) and is thought to result from the T1-shortening effects AVP which is stored in neurosecretory granules of the posterior lobe of the pituitary (19). However, although earlier small studies demonstrated the absence of the bright spot in patients with central DI (20), other larger studies showed that an age-related absence of a the bright spot is observed in up to 52–100% of normal subjects (21). In addition, a loss of the bright spot has also been observed in nephrogenic DI patients bearing AVPR2 mutations (22). Conversely, individual cases with persistent bright spot despite the presence of central DI have also been reported (23). Similarly, we recently showed in a prospective large-scale evaluation that the bright spot was persistent in 36% of patients with central DI, whereas it was missing in 36% of patients diagnosed with primary polydipsia. Consequently, the presence or absence of the bright spot on MRI appears not sensitive and specific enough as a diagnostic test in patients with DI.

### Tests for differential diagnosis

For decades the standard diagnostic test for the evaluation of polyuria polydipsia syndrome was the indirect water deprivation test (4). Here insufficient AVP secretion or effect is diagnosed upon insufficient renal concentration capacity over a prolonged period of water deprivation and its response to exogenous AVP administration. Interpretation of the test results is based on recommendations from Miller *et al*. (4) according to the results of 29 patients with central DI (including 11 patients with partial DI), 2 patients with nephrogenic DI and five patients with primary polydipsia. Patients showing a urinary osmolality below 300 mosm/kg during the water deprivation test are classified as complete central DI if the urinary osmolality increased >50% after exogenous AVP injection. Patients staying below this cut-off are diagnosed as having nephrogenic DI. Patients with partial central DI and primary polydipsia are expected to have a urinary osmolality between 300 and 800 mosm/kg upon thirsting. Urinary osmolality after AVP administration then differentiates patients with partial central DI from patients with primary polydipsia: Patients with partial central DI have an increase in urinary osmolality >9%, whereas patients with primary polydipsia remain below 9%. However, the proposed cut-off levels were *post hoc* derived on a small patient cohort, showed a wide overlap in urinary osmolalities and were never prospectively validated (4).

Consequently, recent evaluations in patients with the polyuria polydipsia syndrome (11, 16) resulted in a diagnostic accuracy of only around 70%, with an especially low accuracy in patients with primary polydipsia.

To overcome these limitations, in 1981, Zerbe *et al*. (24) proposed the ‘direct’ test, with direct measurement of plasma AVP upon osmotic stimulation. Thereby, AVP levels were evaluated in relation to the area of normality describing the physiological relationship between AVP release and plasma osmolality. Plasma AVP levels above the area of normality were defined as nephrogenic DI, levels below as central DI and levels in the normal range were defined as primary polydipsia (7, 24). The results clearly showed that direct measurement of plasma AVP had a superior diagnostic accuracy compared to the classical water deprivation test. However, despite these promising first results disappointing test results derived from recent investigations showing that AVP measurements, especially when using commercially available assays, pointed toward a correct diagnosis in only 38% of patients and were especially bad for differentiation between partial central DI and primary polydipsia (16). Possible reasons are that an accurate definition of the normal physiological relationship describing plasma AVP as a function of osmotic activity has long been missed (25). The precise definition of this normal area, however, is a fundamental prerequisite for the use of direct AVP measurement (16). Secondly, the AVP assay *per se* is subject to several technical limitations, resulting in a high preanalytical instability (1, 26). In addition, the few available reliable assay are not commercially available. Therefore, measurement of AVP never entered every day’s clinical practice.

## Copeptin

**Figure 1:**
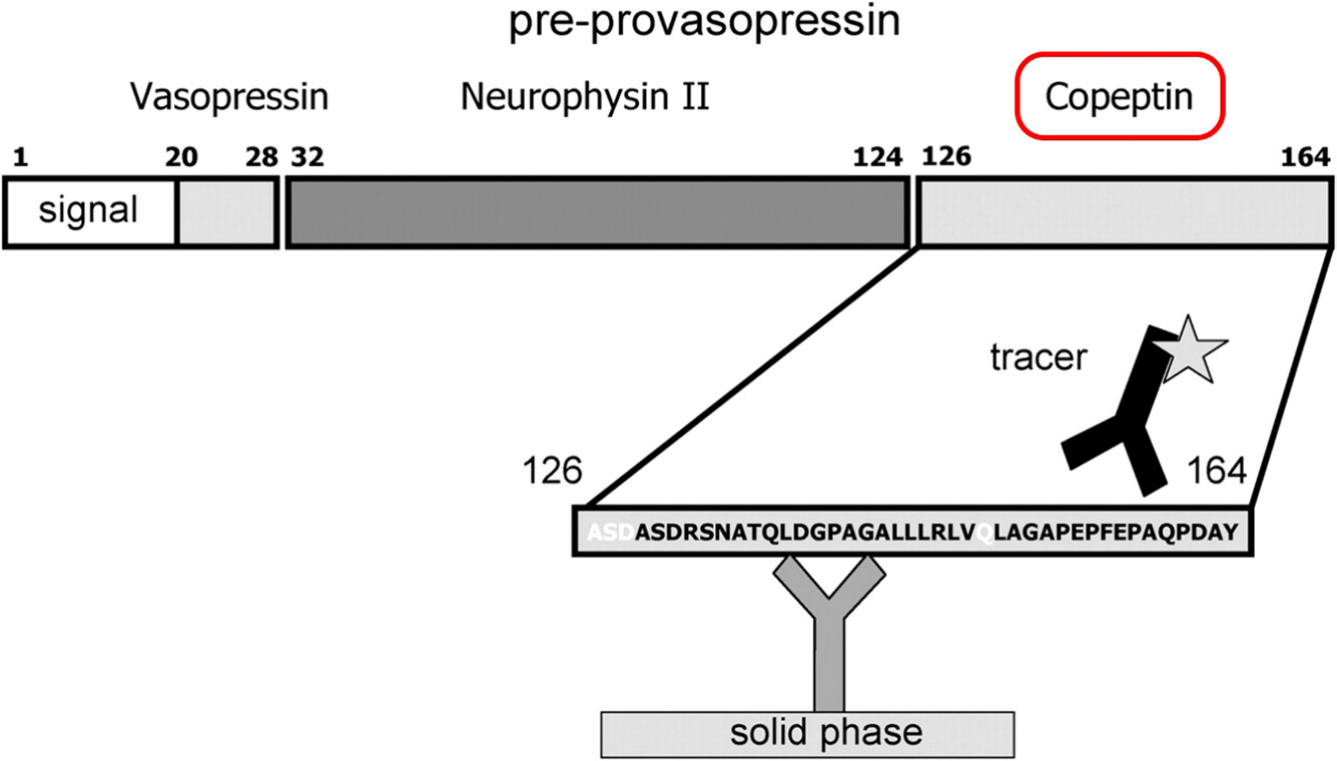
Structure of pre-provasopressin. The prohormone is packaged into neurosecretory granules of magnocellular neurons. During axonal transport of the granules from the hypothalamus to the posterior pituitary, enzymatic cleavage of the prohormone generates the final products: AVP, neurophysin and the COOH-terminal glycoprotein copeptin. Modified from (30) with permission.

Copeptin was detected in 1972 in the posterior pituitary of pigs (27, 28). It derives from the precursor protein pre-pro-vasopressin together with AVP and neurophysin II and is a 39 amino acid long glycosylated peptide with a leucine-rich core region (28, 29). Its molecular mass is around 5 kDa (30) (Fig. 1).

AVP has been characterized as the main hormone for the regulation of body fluid homeostasis and vascular tonus as well as an endocrine stress hormone (31, 32). In contrast, the physiological function of copeptin is largely unknown. It has been proposed to have a role as prolactin-releasing factor, but this could not be confirmed (33, 34). Another function could be the involvement of copeptin in the folding of the AVP precursor, as it seems to interact with the calnexin-calreticulin system in the endoplasmic reticulum (35, 36). Accordingly, a possible relationship between certain forms of familial DI and the inefficient folding of the AVP precursor in the absence of copeptin was proposed (8); however, further examinations are needed. Today, the main use of copeptin is its role as a stable surrogate marker for AVP concentrations in humans (30).

**Figure 2:**
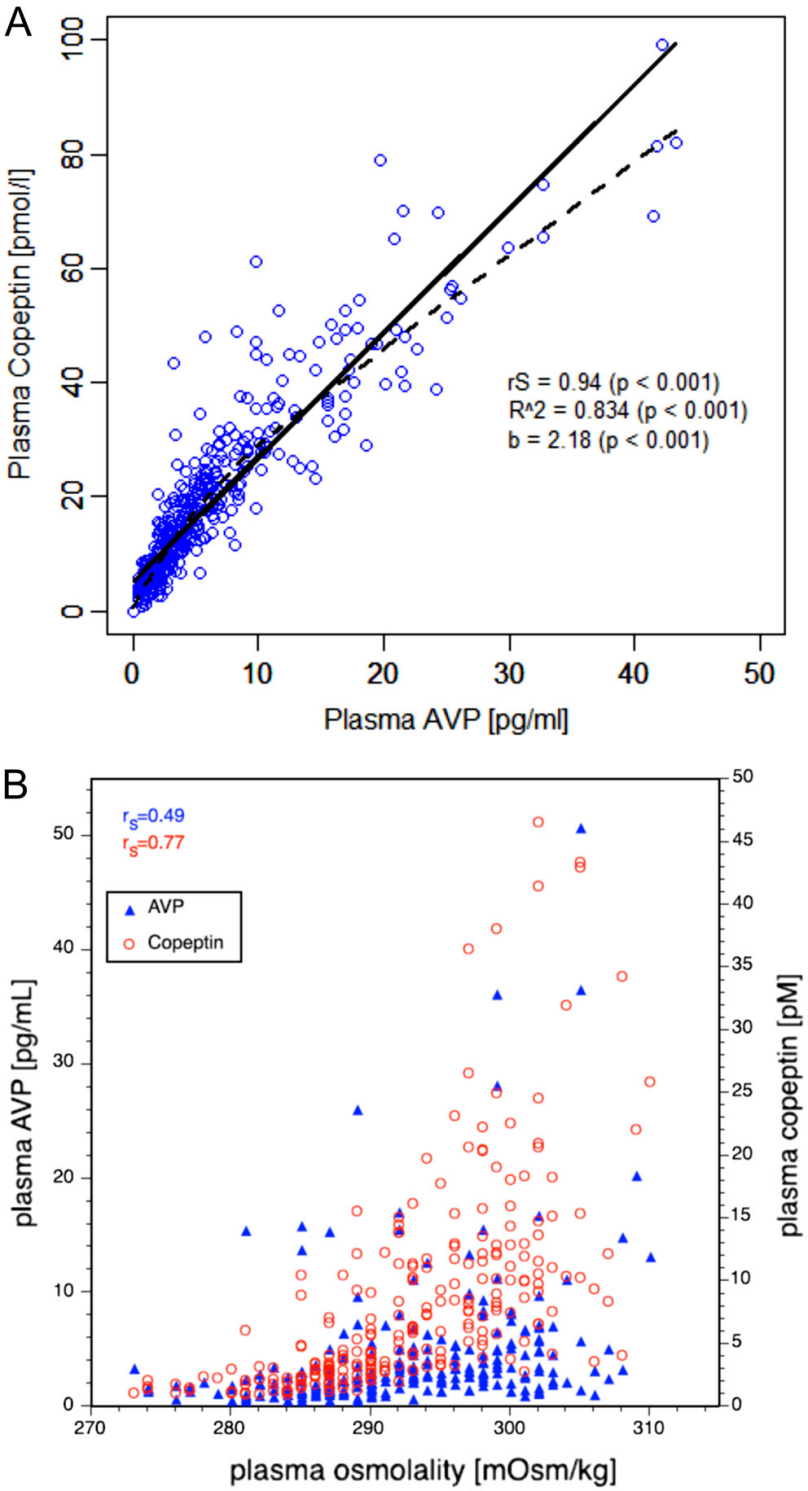
Correlation of arginine vasopressin (AVP) and copeptin (A) and correlation of AVP and copeptin with plasma osmolality (B). Plasma AVP and copeptin concentrations measured during water load and hypertonic saline tests are shown as scatter plot. rS denotes Spearman’s rank correlation coefficients. Modified from (37, 39) with permission.

A strong correlation between plasma AVP (when measured with a well-established radioimmunoassay) and copeptin levels has been demonstrated in healthy volunteers with a correlation index of *r* = 0.8 (37). Notably, the correlation with plasma osmolality was even stronger for copeptin than for AVP (Fig. 2A and B).

The main stimuli for copeptin are similar to AVP, that is an increase in osmolality and a decrease in arterial blood volume and pressure (37, 38). The osmotic trigger of copeptin secretion was first shown in a study with 24 healthy adults, were fluid deprivation as well as hypertonic saline infusion lead to a significant increase in plasma copeptin levels (38). Importantly, we recently showed that copeptin is released in equimolar amounts to AVP in response to osmotic stimulation (with 3% saline infusion), again confirming its high potential as an AVP surrogate for differentiation of osmotic disorders (39).

Conversely, hypotonic saline infusion lead to a prompt suppression of plasma copeptin levels in healthy volunteers (38), similar to an oral water load (37). The prompt response of copeptin secretion upon volume depletion was shown in a baboon model of experimental hemorrhagic shock (40), where plasma copeptin concentration rapidly increased from median levels of 7.5–269 pmol/L, with a subsequent decline to 27 pmol/L after reperfusion.

In addition to osmotic stimulation and arterial pressure, somatic stress as seen in all states of serious illness is also a determinant of copeptin regulation. Several observational studies confirmed the predictive character of plasma copeptin as an unspecific stress marker in various states of acute diseases, including ischemic stroke, myocardial infarction and lower respiratory tract infections (41, 42, 43). We showed that copeptin even more subtly reflects individual stress level than plasma cortisol in patients with increasing disease-related stress (44). Compared to somatic stress, the influence of psychological stress on copeptin release seems to be rather limited, though existent, as shown by recent studies in medical students tested before and after the written exam and upon a psychological stress test (45, 46). Increased levels of plasma copeptin were also reported in response to physical exercise, but levels did not exceed the 99th percentile of the normal range (47, 48).

The half-life of AVP is between 10 and 44 min, depending on the state of hydration, the chosen test method, species and the specificity of immunoassays. We recently showed that copeptin has a two-times-longer half-life in relation to AVP (39). The different half-lives of copeptin and AVP most likely reflect the metabolic clearance rates of these peptides. Other than AVP, which is inactivated by plasma and tissue endopeptidases in the kidney and particularly in the liver (49), the catabolism of copeptin has never been evaluated. The fact that copeptin does not accumulate as a junk protein in the circulation, and that its elimination stops once removed from the circulation, argues against the role of circulating proteases. Theoretically, because of its small size, it could be cleared by the kidneys. In fact, recent data show that copeptin is at least partly eliminated by the kidneys (50) and that in patients with chronic kidney disease, plasma copeptin levels inversely correlate with decreasing glomerular filtration rate suggesting a renal clearance of copeptin (51).

The normal range of plasma copeptin levels has been established in two large clinical trials evaluating healthy volunteers under normo-osmotic conditions. In the first study including 359 subjects, plasma copeptin levels ranged from 1.0 to 13.8 pmol/L with a median concentration of 4.2 pmol/L (30). The second evaluation with over 700 randomly selected volunteers reported comparable results with plasma copeptin levels ranging from 1.0 to 13.0 pmol/L (52). Both studies reported higher median plasma copeptin levels in men than in women; however, no correlation with age was shown (30, 52). Interestingly, the difference between men and women is not present in the hyperosmolar range (39).

While copeptin levels show no significant variation in response to circadian rhythm (53, 54), menstrual cycle (55) or digestion, levels significantly decreased from 4.9 to 3.2 pmol/L even to small amounts of oral fluid intake (200–300 mL) (56), which has to be considered for interpretation of values in clinical practice.

In contrast to AVP, copeptin can be measured in clinical routine with commercially available assays with high-standard technical performance. Two assay are available, on one side the original manual sandwich immunoluminometric assay (LIA) (30) and on the other hand the automated immunofluorescent successor (on the KRYPTOR platform). Advantages of copeptin measurement as opposed to AVP are that it requires only a small sample volume (50 μL of serum or plasma), no extraction step or other preanalytical procedures is needed and that results are usually available in 0.5–2.5 h. Copeptin is much more stable in plasma or serum *ex vivo* with less than 20% loss of recovery for at least 7 days at room temperature and at 14 days at 4°C making the handling of patient blood samples less complicated.

## Copeptin in the differential diagnosis of polyuria polydipsia syndrome

With the development of the copeptin assay, an easy to measure, fast and reliable AVP surrogate with high *ex vivo* stability (30, 37, 57), the focus returned once more to the direct test method.

In a first study, Fenske *et al*. aimed to increase the diagnostic accuracy of the water deprivation test by combining it with copeptin measurement (16). In their cohort of 50 patients with polyuria polydipsia syndrome, baseline plasma copeptin levels >20 pmol/L diagnosed patients with nephrogenic DI, while levels <2.6 pmol/L after an overnight water deprivation test indicated central DI. A ratio of the Δplasma copeptin levels (before and after the water deprivation phase) to the plasma sodium level at the end of the test showed a high diagnostic accuracy of 94% in differentiating patients with central DI from patients with primary polydipsia (16).

In an evaluation of 55 patients with nephrogenic or central DI or primary polydipsia, we described copeptin further as a promising new tool for the diagnosis of polyuria polydipsia syndrome (58). This study confirmed in a larger patient number that patients with nephrogenic DI can be easily diagnosed by using a single baseline copeptin level of >21.4 pmol/L without prior thirsting. Baseline copeptin values in the other entities (i.e. central DI and primary polydipsia) however largely overlapped. Here, we showed that osmotically stimulated copeptin levels of >4.9 pmol/l differentiated patients with central DI from patients with primary polydipsia with a high diagnostic accuracy of 96%. Osmotic stimulation was performed using a standardized combined water deprivation followed by 3% saline infusion test aiming at an increase of plasma sodium levels above 147 mmol/L. The simultaneous evaluation of AVP measurement showed a lower diagnostic differentiation with a diagnostic accuracy of only 80% accuracy, which was especially low for differentiation between partial central DI and primary polydipsia (44%).

We recently validated the copeptin cut-off generated in this study in an international multicenter trial including 156 patients with DI or primary polydipsia (11). In contrast to the studies described above, the test protocol was further simplified in using only the 3% saline infusion without prior thirsting, aiming at a plasma sodium level of at least 150 mmol/L.

Specifically, hypertonic saline was initially given as a bolus dose of 250 mL over 10–15 min, followed by a slower infusion rate of 0.15 mL/kg/min. Serum sodium and osmolality were measured every 30 min and the infusion was terminated once the serum sodium was >150 mmol/L. At this point, copeptin was measured and the patient was asked to drink water at 30 mL/kg within 30 min followed by an intravenous infusion of 5% glucose at 500 mL/h for 1 h. Serum sodium was once more measured after completing the 5% glucose infusion to ensure its return to normal values (11).

**Figure 3:**
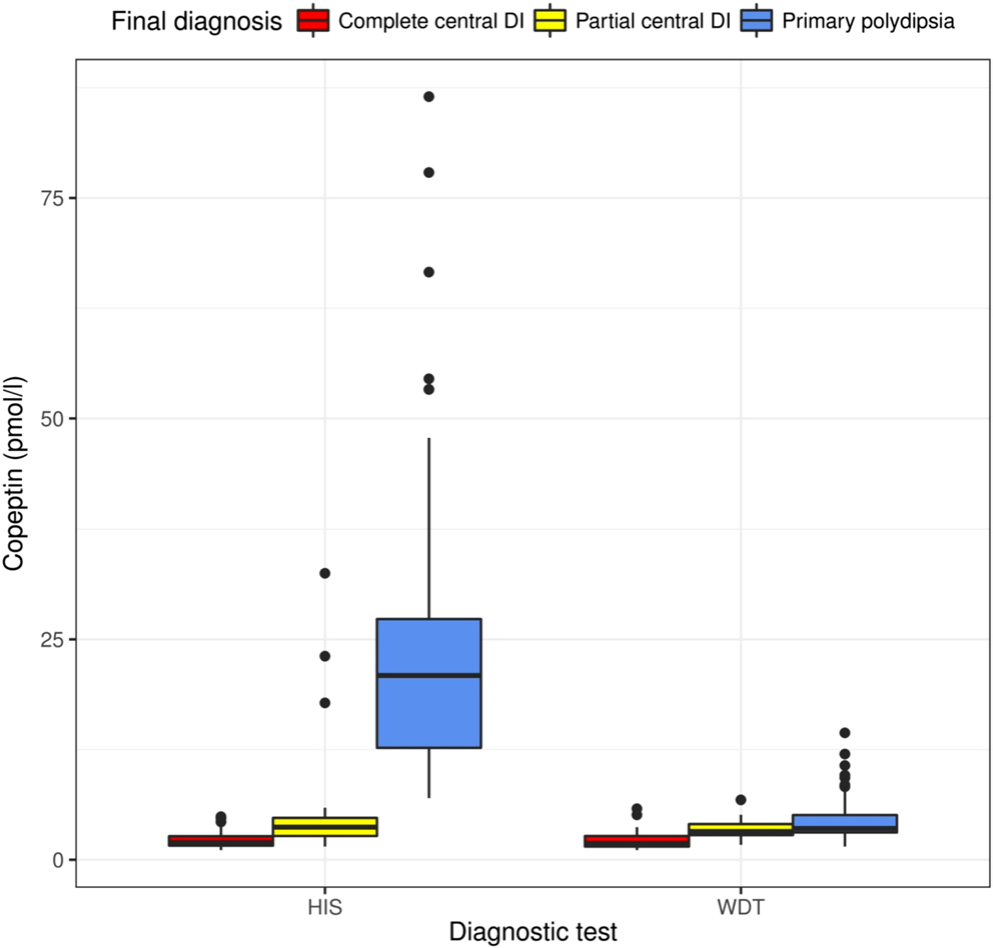
Stimulated copeptin levels in response to the hypertonic saline infusion and water deprivation test in patients with polyuria polydipsia syndrome. Shown are stimulated copeptin levels in response to the hypertonic saline infusion test (HIS) and water deprivation test (WDT) in patients with polyuria polydipsia syndrome that was caused by complete central diabetes insipidus (DI) or partial central diabetes insipidus as compared with primary polydipsia. The horizontal line in each box represents the median, the lower and upper boundaries of the boxes the interquartile range, the ends of the whisker lines the minimum and maximum values within 1.5 times the interquartile range and the dots outliers. Modified from (11) with permission.

Under this osmotic stimulation, 97% of the patients were correctly diagnosed using the copeptin cut-off level of >4.9 pmol/L (Fig. 3). The diagnostic accuracy was similarly accurate in distinguishing patients with partial DI from patients with primary polydipsia with a correct diagnosis in 95%. Again, copeptin levels of all three included patients with nephrogenic DI exceeded the cut-off level of 21.4 pmol/L. Contrary to the study of Fenske described earlier, the proposed copeptin-sodium ratio did not improve the diagnostic accuracy of the water deprivation test (16), resulting in a diagnostic accuracy of only 44% (11). The proposed copeptin cut-off level of <2.6 pmol/L after an overnight water deprivation test to diagnose complete central DI had a diagnostic accuracy of 78%. The fact that the determination of copeptin after water deprivation alone does not lead to an improved diagnostic accuracy is most likely due to the inadequate osmotic stimulation. This observation is confirmed by the fact that most patients in the study did not reach hyperosmotic plasma sodium levels during the classical water deprivation test.

**Figure 4:**
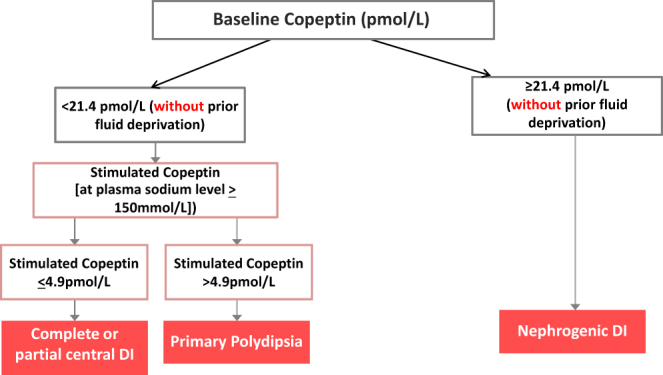
New algorithm for the differential diagnosis of polyuria polydipsia syndrome. Modified from (2).

Accordingly, osmotic stimulation by hypertonic saline solution is needed to obtain reliable copeptin measurements. It is however important to note that hypertonic saline infusion requires close monitoring of sodium levels to ensure increase of plasma sodium levels into the hyperosmotic range (3, 59) while preventing osmotic overstimulation (11). In settings where regular and rapid sodium monitoring is not possible, the hypertonic saline test should not be performed. Also, it might be prudent for clinical practice to aim at a plasma sodium level >147 mmol/L instead of 150 mmol/L. Rapid normalization of sodium levels after the osmotic stimulation is also crucial to guarantee the safety of the test (11). Based on these results, it was concluded that the hypertonic saline test plus copeptin measurement might replace the classical water deprivation test in the future differential diagnosis of hypotonic polyuria (60). The recommended new algorithm for the differential diagnosis of polyuria polydipsia syndrome is displayed in Fig. 4.

Importantly, the majority of patients preferred the hypertonic saline stimulation with copeptin measurement over the classical water deprivation test, although side effects were slightly more common in the hypertonic saline test phase. Most probably, the reason was the much shorter test duration (approximately 2 h for the hypertonic saline test vs 17 h for the water deprivation test) (11).

In conclusion, copeptin is a valuable and reliable diagnostic marker in the differential diagnosis of polyuria polydipsia syndrome. In a patient with unclear hypotonic polyuria and polydipsia, determination of basal copeptin levels is recommended to exclude nephrogenic DI. In patients with high suspicion of complete central DI, an overnight water deprivation test might confirm diagnosis provided urine osmolality remains below 300 mosm/kg and plasma sodium levels increase above 147 mmol/L. In all other patients, copeptin measurement after osmotic stimulation with 3% saline solution aiming at a plasma sodium level above 147 mmol/L is recommended.

Importantly, close monitoring of plasma sodium levels is needed to ascertain a diagnostically meaningful increase in plasma sodium within the hyperosmotic range while preventing a marked increase, to which females appear to be more vulnerable than males (11).

## Copeptin in the diagnosis of postsurgical DI

An additional characteristic of copeptin is its use as a predictive marker for the development of postoperative DI after pituitary surgery. In a first proof-of-concept study in 2007, we used the insulin tolerance test and showed that copeptin measured during insulin-induced hypoglycemia remains low in patients with DI while increasing in patients with intact posterior pituitary function. Specifically, a copeptin level <4.75 pmol/L identified patients with complete central DI at 3 months after transsphenoidal pituitary surgery with a high accuracy (61).

**Figure 5:**
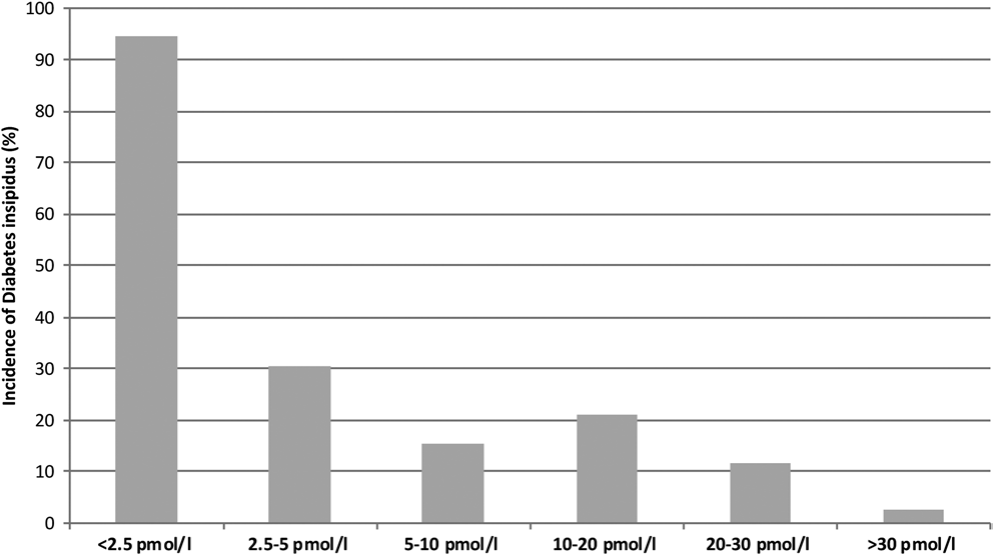
Copeptin levels upon pituitary surgery. A copeptin cut-off level of <2.5 pmol/L, measured within the first 12 h after surgery, had a positive predictive value for development of postoperative diabetes insipidus of 81% and a specificity of 97%; while a level >30 pmol/L excluded it with a negative predictive value of 95% and a sensitivity of 94%. Modified from (69) with permission.

However, hypoglycemic stimulated copeptin is not feasible as a routine postoperative test for DI as it may be associated with severe hypoglycemia which is contraindicated in patients with cardiovascular disease or seizure history as well as during immediate postoperative recovery. In fact, surgery itself is a stressful event known to stimulate hypothalamic stress hormone release including AVP (62). We therefore hypothesized that pituitary surgery itself can be used as a ‘stress test’ to assess the functionality of AVP and copeptin secretion. We performed a prospective multicenter trial including 205 patients undergoing pituitary surgery. The 24% who developed central DI had significantly lower copeptin levels on the first postoperative day compared to patients with an uneventful course. The *post hoc* derived copeptin cut-off level of <2.5 pmol/L had a positive predictive value for development of postoperative DI of 81% and a specificity of 97%, while a level >30 pmol/L excluded it with a negative predictive value of 95% and a sensitivity of 94% (Fig. 5). Accordingly, copeptin measurement after pituitary surgery is helpful to predict the onset of central DI and may therefore be a novel marker for early goal-directed management of postoperative DI.

## Conclusions and future prospects

In conclusion, the availability of copeptin has led to a ‘revival’ of the direct test in the differential diagnosis of the polyuria polydipsia syndrome. Copeptin is a stable surrogate marker of AVP able to identify patients with nephrogenic DI with a baseline measurement without prior thirsting. For the differentiation of central DI and primary polydipsia, osmotic stimulation with hypertonic saline is needed, with a copeptin cut-off of 4.9 pmol/L differentiating these two entities with a high diagnostic accuracy.

To further simplify the algorithm, an alternative stimulation of the neurohypophysis without the necessity of constant monitoring of plasma sodium levels would be preferable. Arginine infusion is known to stimulate various hormones secreted by the anterior pituitary gland such as growth hormone and prolactin (63, 64). It is therefore used as a standard test in the evaluation of suspected growth hormone deficiency in children and adult patients (65, 66, 67). We therefore hypothesized that arginine would also be a stimulus for copeptin secretion and could differentiate between central DI and primary polydipsia. Indeed, our recent data in patients with central DI or primary polydipsia (68) showed that arginine is a potent stimulator of the neurohypophysis with consecutive copeptin secretion. Measurement of plasma copeptin levels after arginine infusion was able to differentiate patients with primary polydipsia from patients with central DI with a high diagnostic accuracy. These findings are particularly notable with regard to the generally good tolerability of the arginine infusion and short test duration of 2 h. Therefore, in the future, arginine stimulated copeptin measurement could further simplify the differential diagnosis of polyuria polydipsia syndrome.

## Declaration of interest

M C C received speaker honoraria from Thermofisher AG, the manufacturer of the Copeptin assay.

## Funding

M Christ-Crain was supported by a grant from the Swiss National Science Foundation (SNF-162608) and the University Hospital Basel, Switzerland.
